# Platon: identification and characterization of bacterial plasmid contigs in short-read draft assemblies exploiting protein sequence-based replicon distribution scores

**DOI:** 10.1099/mgen.0.000398

**Published:** 2020-06-24

**Authors:** Oliver Schwengers, Patrick Barth, Linda Falgenhauer, Torsten Hain, Trinad Chakraborty, Alexander Goesmann

**Affiliations:** ^1^​ Bioinformatics and Systems Biology, Justus Liebig University Giessen, Giessen, Germany; ^2^​ Institute of Medical Microbiology, Justus Liebig University Giessen, Giessen, Germany; ^3^​ German Center for Infection Research (DZIF), partner site Giessen-Marburg-Langen, Giessen, Germany; ^‡^​ Present address: Institute of Hygiene and Environmental Health, Justus Liebig University, Giessen, Germany

**Keywords:** bacteria, plasmids, NGS, whole-genome sequencing

## Abstract

Plasmids are extrachromosomal genetic elements that replicate independently of the chromosome and play a vital role in the environmental adaptation of bacteria. Due to potential mobilization or conjugation capabilities, plasmids are important genetic vehicles for antimicrobial resistance genes and virulence factors with huge and increasing clinical implications. They are therefore subject to large genomic studies within the scientific community worldwide. As a result of rapidly improving next-generation sequencing methods, the quantity of sequenced bacterial genomes is constantly increasing, in turn raising the need for specialized tools to (i) extract plasmid sequences from draft assemblies, (ii) derive their origin and distribution, and (iii) further investigate their genetic repertoire. Recently, several bioinformatic methods and tools have emerged to tackle this issue; however, a combination of high sensitivity and specificity in plasmid sequence identification is rarely achieved in a taxon-independent manner. In addition, many software tools are not appropriate for large high-throughput analyses or cannot be included in existing software pipelines due to their technical design or software implementation. In this study, we investigated differences in the replicon distributions of protein-coding genes on a large scale as a new approach to distinguish plasmid-borne from chromosome-borne contigs. We defined and computed statistical discrimination thresholds for a new metric: the replicon distribution score (RDS), which achieved an accuracy of 96.6 %. The final performance was further improved by the combination of the RDS metric with heuristics exploiting several plasmid-specific higher-level contig characterizations. We implemented this workflow in a new high-throughput taxon-independent bioinformatics software tool called Platon for the recruitment and characterization of plasmid-borne contigs from short-read draft assemblies. Compared to PlasFlow, Platon achieved a higher accuracy (97.5 %) and more balanced predictions (F1=82.6 %) tested on a broad range of bacterial taxa and better or equal performance against the targeted tools PlasmidFinder and PlaScope on sequenced *Escherichia coli* isolates. Platon is available at: http://platon.computational.bio/.

## Data Summary

1. Platon was developed as a Python 3 command line application for Linux.
2. The complete source code and documentation are available on GitHub under a GPL3 license: https://github.com/oschwengers/platon and http://platon.computational.bio.
3. All database versions are hosted at Zenodo (DOI: 10.5281/zenodo.3349651).
4. Platon is available via the bioconda package platon.
5. Platon is available via the PyPI package cb-platon.
6. The bacterial representative sequences for UniProt’s UniRef90 protein clusters, complete bacterial genome sequences from the National Center for Biotechnology Information (NCBI) RefSeq database, complete plasmid sequences from the NCBI genomes plasmid section, created artificial contigs, replicon distribution score (RDS) threshold metrics and raw protein replicon hit counts used to create and evaluate the marker protein sequence database are hosted at Zenodo (DOI: 10.5281/zenodo.3759169).
7. Twenty-four *Escherichia coli* isolates sequenced with short-read (Illumina MiSeq) and long-read sequencing technologies (Oxford Nanopore Technology GridION platform) used for real data benchmarks are available under the following NCBI BioProjects: PRJNA505407 and PRJNA387731.

**Impact Statement**

Plasmids play a vital role in the spread of antibiotic resistance and pathogenicity genes. The increasing numbers of clinical outbreaks involving resistant pathogens worldwide pushed the scientific community to increase their efforts to comprehensively investigate bacterial genomes. Due to the maturation of next-generation sequencing technologies, entire bacterial genomes, including plasmids, are now sequenced on a huge scale. To analyse draft assemblies, a mandatory first step is to separate plasmid from chromosome contigs. Recently, many bioinformatic tools have emerged to tackle this issue. Unfortunately, several tools are only implemented as interactive or web-based tools, making them unavailable for the necessary high-throughput analysis of large datasets. Other tools providing such a high-throughput implementation, however, often come with certain drawbacks, e.g. providing taxon-specific databases only, not providing actionable, i*.*e. true, binary classification, or showing classification performance that is biased towards either sensitivity or specificity. Here, we introduce the tool Platon, implementing a new replicon distribution-based approach combined with higher-level contig characterizations to address the aforementioned issues. In addition to the plasmid detection within draft assemblies, Platon provides the user with valuable information on certain higher-level contig characterizations. We show that Platon provides a balanced classification performance as well as a scalable implementation for high-throughput analyses. We therefore consider Platon to be a powerful, species-independent and flexible tool to scan large quantities of bacterial whole-genome sequencing data for their plasmid content.

## Introduction

Plasmids are bacterial extrachromosomal DNA elements that replicate independently of the chromosome. They are mostly circular, have characteristic copy numbers per cell and carry genes that are usually not essential under normal conditions but rather allow bacteria to adapt to specific environments and conditions [1]. These genes, for instance, provide antibiotic or heavy metal resistance, are involved in alternative metabolic pathways or encode for virulence factors [2]. As plasmids are not only inherited by daughter cells, but can also be dispersed by horizontal gene transfer, they can spread rapidly within and between bacterial populations [3–5]. For example, identical antibiotic resistance plasmids have already been isolated from humans and animals [6]. Thus, plasmids are important mediators of antibiotic resistance spread and recent findings have confirmed that they frequently play a major role in clinical outbreaks [7, 8]. Therefore, it is of huge importance to properly identify and analyse plasmids.

Such analysis can be performed by plasmid DNA isolation followed by sequencing [9]. However, due to decreased sequencing costs, it is now affordable and often easier to sequence the entire genome of bacterial organisms using next-generation whole-genome shotgun sequencing [10]. Furthermore, this approach allows the reanalysis of already sequenced genomes to identify plasmids that have not been detected before. Unfortunately, this introduces a new issue that needs to be addressed: plasmid and chromosomal contigs are mixed in draft assemblies and need to be distinguished from each other.

This task, however, is hard to achieve for biological and technical reasons [11]. Plasmids often contain mobile genetic elements, e.g. transposons and integrons, which are drivers for the genetic exchange between different DNA replicons and regions [12, 13]. Hence, these genetic elements are often encoded on both replicon types and thus the origin of DNA fragments encoding such elements is hard to predict. Modern short-read assemblers add additional intricacy, further aggravating these issues, as they are notoriously hard pressed to correctly assemble repetitive regions such as the aforementioned DNA elements [14]. To tackle this issue, many new bioinformatic tools have recently been developed, following different approaches: (i) Recycler and plasmidSPAdes [15, 16] exploit coverage variations of sequenced DNA fragments within a genome; (ii) PLACNET investigates paired-end reads linking contig ends [17]; (iii) PlasmidFinder searches for plasmid specific motifs, i.e. incompatibility groups [18]; (iv) cBar, PlasFlow and mlPlasmids use machine learning methods to classify k-mer frequencies [19–21]; (v) PlaScope and PlasmidSeeker perform fast k-mer-based database searches of known plasmid sequences [22, 23]; Recycler additionally exploits information on circularization [15]. Overall, each approach provides unique advantages and drawbacks. For example, approaches based on sequencing coverage variations are unable to detect plasmids with copy numbers equal to the chromosome, whereas sequence motif- and k-mer-based methods tend to identify only known plasmids. This often leads to distinct profiles in terms of sensitivity and specificity, which are often biased towards one of the metrics, and as this impacts on the conducted analysis a choice must be made between conservative or more aggressive classifications [11].

A further aspect of growing importance is ‘big data’ awareness. Due to increasing quantities of generated sequence data [24], there is a growing need for automated high-throughput analysis tools. Unfortunately, not all of the currently available bioinformatics software tools are suitable for high-throughput analysis, let alone technical integration into larger analysis pipelines [25–27] due to interactive designs or web-based implementations [17, 18, 21, 28]. Taxon-specific database designs also pose additional barriers, as users might not have sufficient computational resources or bioinformatics support to build customized or large multi-taxon databases [20, 22]. Furthermore, dependence on raw or intermediate data such as sequence reads and assembly graphs might impede analyses, as such data might not be available [15, 16]. In order to allow for big data scaling necessities, bioinformatic software tools should therefore be designed and implemented in a high-throughput savvy manner, including: (i) where possible a taxon-independent database design; (ii) a non-interactive command line implementation; and (iii) an actionable classification output, i.e. a true binary classification.

To address these issues we present Platon, a new bioinformatics software tool to distinguish and characterize plasmid contigs from chromosome contigs in bacterial draft assemblies following a new approach: analysis of the replicon distribution differences of protein-coding genes, i.e. frequency differences for being encoded on plasmid or chromosome contigs. The rationale behind this protein sequence-based replicon, i.e. chromosome vs plasmid, classification is a natural distribution bias of certain protein-coding genes. For instance, essential housekeeping genes that are mandatory for bacterial organisms are mostly encoded on chromosomes [2]. In contrast, genes providing an evolutionary advantage under distinct situations are quite widespread on plasmids, e.g. antibiotic resistance and virulence genes. Here, we introduce the replicon distribution score (RDS), a new metric to express the measured bias of protein-coding genes’ replicon distributions to distinguish plasmid- from chromosome-related contigs.

## Methods

### Marker protein sequences and computation of replicon distribution scores

To build a database of marker protein sequences (MPSs) we collected all bacterial representative sequences of UniProt’s UniRef90 protein clusters (*n*=69 803 841) [29] and analysed their replicon distributions, i.e. the normalized plasmid vs chromosome abundance ratios. Therefore, we conducted a homology search via Diamond [30] of all MPS against coding sequences (CDSs) predicted via Prodigal [29] on two reference replicon sets, i.e. all National Center for Biotechnology Information (NCBI) plasmid sequences from the bacterial NCBI Genomes plasmid section (*n*=17 369) (ftp://ftp.ncbi.nlm.nih.gov/genomes/GENOME_REPORTS/plasmids.txt) and the chromosomes of all complete bacterial NCBI RefSeq release 98 genomes. To prevent potential plasmid contamination in the chromosome set, all replicons shorter than 100 kbp were excluded, resulting in 17 430 chromosome sequences. The resulting alignment hit counts (*A*) of the single best hit per sequence with a sequence coverage ≥80 % and a sequence identity of at least 90 %, as well as the number of replicons (*R*) for both plasmids (*p*) and chromosomes (*c*) were integrated into a normalized, transformed and scaled RDS for each cluster, defined by:

$$RDS=2*\left(\frac{F_{p}}{\left(F_{p}+F_{c}\right)}-0.5\right)*\frac{\left|F_{p}-F_{c}\right|}{\varphi }*\left(1-P_{val}\right)$$

with $F_{p}=\frac{A_{p}}{R_{p}}$, $F_{c}=\frac{A_{c}}{R_{c}}$, $\varphi =\frac{\sum_{i=1}^{n}\left|F_{p,i}-F_{c,i}\right|}{n}$, where *n* is the number of elements of the MPS database and *p*_val_ is the *P* value of a two-sided Fisher’s exact test using a contingency table of hit and no-hit counts for both replicon types.

Thus, the RDS value of a protein sequence represents its replicon distribution bias as both the ratio and the absolute difference of hit count frequencies as well as its statistical power. As a first factor of the formula, the hit count frequency ratio $\left(\frac{F_{p}}{\left(F_{p}+F_{c}\right)}\right)$ is transformed by the minuend −0.5 and the factor 2 to the range [−1,1] and hence, shifts the RDS values of chromosomal proteins to a negative range [−1,0] and to a positive value range [0,1] for proteins with a positive plasmid bias. To integrate the scale of the difference in the hit count frequencies, we added the absolute difference of replicon hit count frequencies ($F_{p}-F_{c}$) normalized to the mean difference of hit count frequencies of all MPSs (φ) as a second factor. In order to also include a measure of statistical confidence in the new RDS metric, a third factor ($1-p_{val}$) was added, taking the *P* value of a two-sided Fisher’s exact test using a contingency table of hit and non-hit counts of both replicon types under the assumption that these are not equally distributed – the main idea behind the RDS metric. Thus, RDS values resulting from statistically insignificant hit count numbers are minimized towards zero. In order to finally classify entire contigs, the mean RDS of all the per-protein-sequence RDS values of each contig is calculated and then tested against defined thresholds. Predicted CDSs, for which no MPS can be identified are assigned the neutral default RDS value of zero.

### Evaluation of replicon distribution scores

In order to assess the discriminative power of protein sequence based RDS, we created 10 random fragments of each sequence in the reference replicon sets for each of the following lengths: 1, 5, 10, 20 and 50 kbp. For each random fragment, a mean RDS was computed and tested against a range of discrimination thresholds between −50 and 10 with a step size of 0.1. For each discrimination threshold, a confusion matrix was set up upon which sensitivity [*tp/(tp+fn*)], specificity [(*tn/(tn+fp*)] and accuracy [(*tp+tn)/(tp+tn+fp+fn*)] metrics were calculated, where *tp*, *tn*, *fp* and *fn* are the number of true positives, true negatives, false positives and false negatives, respectively.

### Higher-level contig characterization

The comprehensive characterization of contigs by higher-level plasmid-related sequence analysis often requires many specialized command line and web-based tools and thus is a time-consuming task. To streamline this process, we implemented and included many higher-level sequence analyses in the workflow. Hence, Platon provides valuable contig information and can take advantage thereof by integrating applied heuristics into the classification process. Contigs are comprehensively characterized using different approaches: (i) testing for circularization; (ii) detection of incompatibility groups; (iii) detection of rRNA genes; (iv) detection of antimicrobial resistance genes; (v) homology search against reference plasmid sequences; (vi) detection of oriT sequences; (vii) detection of plasmid replication genes; (viii) detection of mobilization genes; (ix) detection of conjugation genes.

Contigs are tested for circularization by aligning sub-sequences from both ends against each other using nucmer from the MUMmer package [31]. Contig ends with overlaps larger than or equal to 100 bp and an identity >95 % are considered to be circularizable. To detect incompatibility groups, Platon conducts a homology search using the PlasmidFinder database (*n*=273) [18] via blast+ [32] against contigs filtering for query coverages ≥60 % and percentage sequence identities >90 %. Although rare exceptional cases are described in the literature [33], the majority of ribosomal genes are encoded on chromosomes [33]. In order to exploit this distribution bias, ribosomal genes are detected via Rfam and Infernal [34]. As antimicrobial resistance genes are often encoded on mobile genetic elements (e.g. plasmids), Platon uses the NCBI ResFam hidden Markov models (HMM) database [35] and HMMER [36] to detect potential antimicrobial resistance genes. In order to detect contigs as sub-sequences of larger plasmids or entire plasmids with known sequences, Platon conducts a homology search via blast+ [30] against the RefSeq plasmid sequence database [37] filtering for query coverages and percentage sequence identities ≥80 %, setting a dynamic *-word_size* parameter to 1 % of the query contig length. To detect oriT sequences, Platon conducts a blast+ [32] homology search against oriT sequences of the MOB suite database [38] filtering for both 90 % sequence coverage and identity.

Depending on their genetic backbone, plasmids can be mobilizable or conjugative [4]. The presence or absence of specialized proteins involved in the replication, mobilization and conjugation processes plays an important role as a determinant for the classification of plasmids. Platon takes advantage of the highly plasmid-specific nature of these proteins by scanning predicted CDSs against a custom HMM database. Therefore, we extracted relevant RefSeq PCLA protein clusters via text mining and subsequently built HMM models on aligned protein sequences per cluster (Table S1, available with the online version of this article), creating two distinct HMM databases: replication and conjugation, comprising 257 and 1 663 HMM models, respectively. To take advantage of the expert knowledge and manual efforts that led to the high-quality relaxase HMM profiles of the MOBscan database [39], these were incorporated into this workflow. A scan against each HMM database is integrated into the classification process.

### Platon analysis workflow

**Fig. 1.**
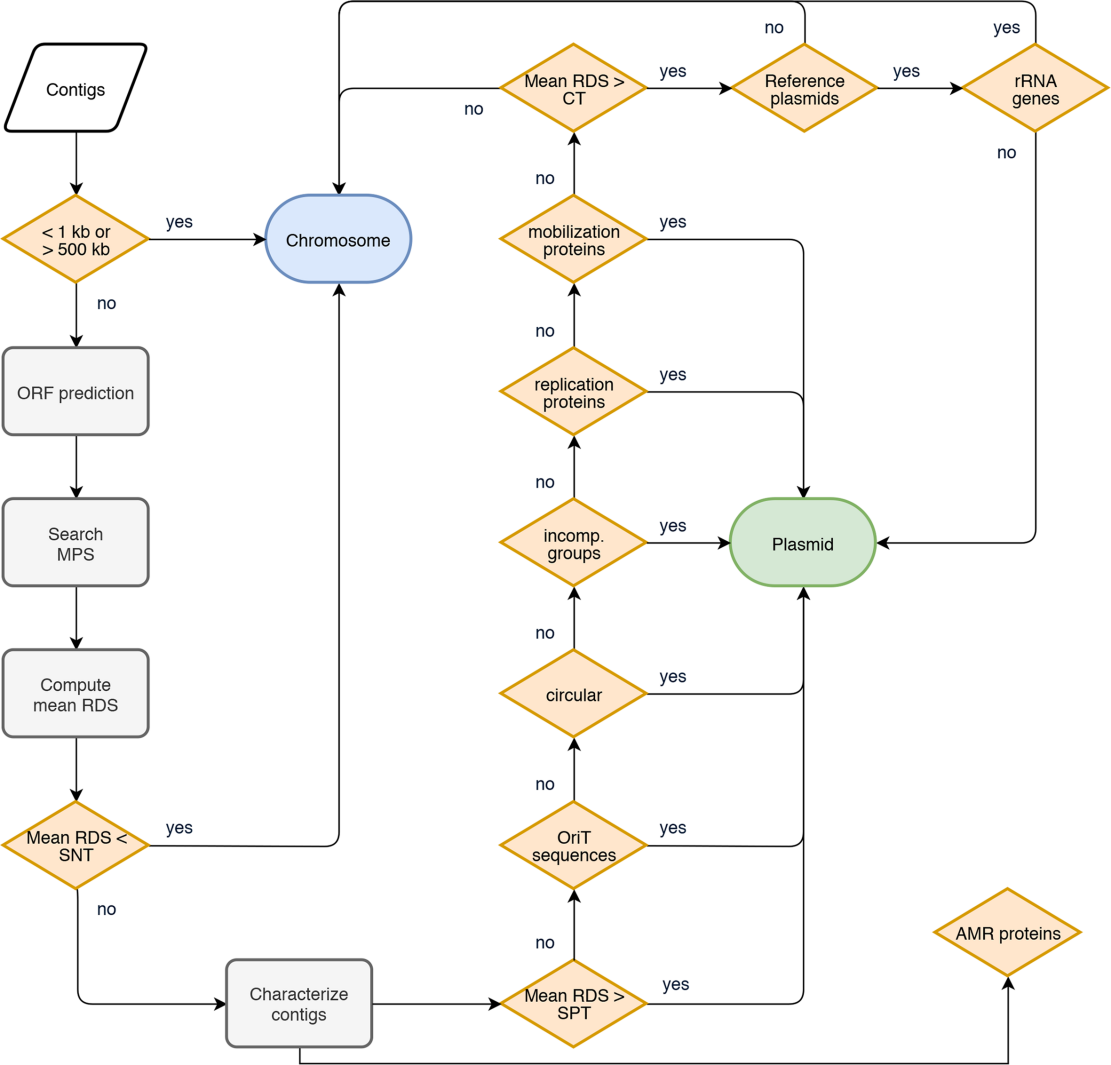
Flowchart describing the workflow implemented in Platon. ORF, open reading frames; MPS, marker protein sequence; RDS, replicon distribution score; SNT, sensitivity threshold; SPT, specificity threshold; incomp. groups, incompatibility groups; CT, conservative threshold.

Platon combines the analysis of the replicon distribution bias of protein sequences with a set of higher-level contig characterizations to predict the replicon origin of contigs (Fig. 1). In a first step, Platon classifies all contigs with a length smaller than 1 kbp or larger than 500 kbp as chromosomal. The rationale behind this heuristic is that sequences with <1 kbp seldom host either a CDS or other exploitable information that would permit reliable classification. On the other hand, from our experience, contigs >500 kbp rarely or never originate from plasmids, as those often encode genetic features hindering the assembly of larger sequences, for example transposons and integrons. Thus, this heuristic enhances the overall analysis runtime performance without unduly sacrificing classification performance.

In a second step, CDSs are predicted via Prodigal [40] and searched against a database of MPS via Diamond [30], applying rigorous detection cutoffs in line with the cluster specifications of the underlying UniRef90 clusters, i.e. a coverage of at least 80 % and a sequence identity of at least 90 %. For each contig, the mean RDS of all detected MPSs is computed. Contigs with a mean RDS lower than the sensitivity threshold (SNT) are classified as chromosomal sequences. The remaining contigs are then comprehensively characterized as described in the previous section.

Contigs are subsequently classified as plasmid sequences if one or more of the following conditions are met: the contig (i) has a mean RDS larger than the specificity threshold (SPT); (ii) can be circularized; (iii) provides at least one replication or mobilization protein; (iv) contains an incompatibility factor; (v) contains an oriT sequence; (vi) has a mean RDS larger than the conservative threshold (CT) and a blast+ [32] hit against the RefSeq plasmid database without encoding ribosomal genes.

### Performance benchmarks

The overall replicon classification performance of Platon v1.3.1 was benchmarked against PlaScope 1.3.1, PlasFlow 1.1.0 and the PlasmidFinder database (version 2018-11-20) in two setups: a targeted benchmark comparing Platon against PlaScope and PlasmidFinder on sequenced *Escherichia coli* isolates and an untargeted benchmark comparing Platon against PlasFlow on simulated short-read assemblies of all complete RefSeq genomes. PlaScope and PlasFlow were used with default parameters and publicly provided prebuilt databases. As PlasmidFinder is currently only available as a web tool or via Docker, which is not usable in our HPC cluster setup, its workflow was reimplemented in bash using equal blast+ parameters (*-perc_identity 90*; query coverage >=60 %). As both PlaScope and PlasFlow allow a third classification label, i.e*.* unclassified, and thus are not true binary classifiers, replicon fragments were treated as being classified as chromosomes as long as they were not explicitly classified as plasmid for the sake of comparability. For each benchmark, we calculated sensitivity, specificity and accuracy metrics as described above. To also include statistically balanced metrics, we calculated the positive predictive power (PPV) [*tp/(tp+fp*)], the negative predictive power (NPP) [*tn/(tn +fn*)] as well as F1 score and Matthews correlation coefficient (MCC) using the SciKit-learn Python package. For the simulated benchmark dataset, we used all bacterial NCBI RefSeq genomes (release 98) at the assembly level ‘Complete Genome’ (*n*=13 930) to generate artificial short reads via ART (2.5.8) [41] with read lengths of 150 bp, 40-fold coverage, 500 bp mean insert size and 10 bp insert size standard deviation. Simulated reads were then assembled with SPAdes (3.12.0) [42] using the *--careful* and *--cov-cutoff auto* parameters. The resulting contigs (*n*=820 932) were aligned against original genomes with blast+ (2.7.1) [32] and finally labelled either as chromosome or plasmid according to the single best blast+ hit.

To benchmark on real data, we isolated 24 multidrug-resistant *E. coli* genomes in Germany from humans, dogs and horses [43] (Table S2). Isolates were sequenced on the Illumina MiSeq platform using the Nextera XT sequencing kit (2×250 or 2×300 nt) as well as the Oxford Nanopore GridION platform using a SpotON Mk I R9 version flow cell (FLO‐MIN106), native barcoding kit (EXP-NBD103) and 1D chemistry (SQK-LSK108). Oxford Nanopore raw data (fast5) were basecalled using Albacore (1.11.8) (https://community.nanoporetech.com). For each isolate, two assemblies were performed: (i) a hybrid assembly using Unicycler v0.4.6 [44] and (ii) a short read-only assembly with SPAdes. For 21 isolates, the hybrid assembly resulted in circular chromosomes, which were used as the benchmarking ground truth, as the majority of remaining contigs thus originate from unclosed plasmids. The remaining three isolates with unclosed chromosomes were excluded from the benchmark dataset, as the former requirement was not fulfilled. Short-read contigs <1 kbp were discarded. The remaining contigs (*n*=1 337) were then aligned against closed hybrid assemblies as described above. The raw sequencing data for all 24 isolates are available as NCBI BioProjects (PRJNA505407, PRJNA387731).

## Results and discussion

### Creation of the MPS database and RDS-based inference of contig origins

**Fig. 2.**
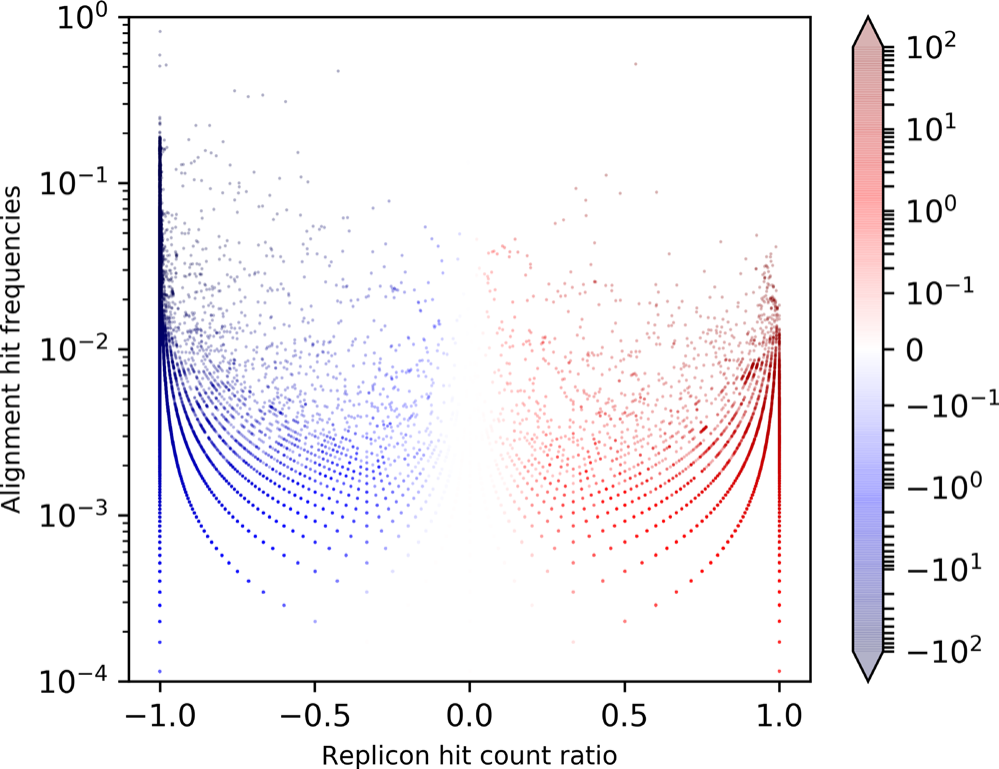
Replicon distribution and alignment hit frequencies of marker protein sequences. Shown here are summed plasmid and chromosome alignment hit frequencies per marker protein sequence plotted against plasmid/chromosome hit count ratios scaled to [−1, 1]; Hue: normalized replicon distribution score values (min=−100, max=100), hit count outliers below 10^−4^ and above 1 are discarded for the sake of readability.

The proposed new metric RDS exploits the natural distribution biases of protein-coding genes between chromosomes and plasmids to classify the origin of contigs from short-read assemblies. In order to investigate and test this rationale, we aligned a broad range of bacterial protein sequences (*n*=69 803 841) from UniProt’s UniRef90 protein cluster representative sequences against a set of known chromosome and plasmid reference replicons from the NCBI RefSeq and NCBI Genomes databases and 12 795 544 of these protein sequences could be aligned to at least 1 replicon. For each of these protein sequences, a two-sided Fisher’s exact test was conducted and sequences with a *P* value of 1 were excluded. The remaining protein sequences (*n*=4 108 727), along with their RDS values, product description and sequence lengths, were then used to compile the final MPS database. For 99.5 % of these protein sequences (*n*=4 089 068) a transformed hit count ratio smaller than −0.5 (*n*=3 600 927) or larger than 0.5 (*n*=488 141) was computed, indicating a rather unequal distribution between chromosomes and plasmids (Fig. 2). However, only a minor fraction of 7.8 % (*n*=322 151) of all MPSs had a normalized alignment hit count sum regarding both replicon types larger than 0.001. Hence, the majority of MPS database sequences were relatively rarely detected on average. These findings endorse the incorporation of the statistical significance of each MPS replicon distribution as well as the scaling by the absolute difference of replicon hit count frequencies in order to raise the contribution of abundant protein sequences and decrease the contribution of rare protein sequences, for which insufficient data are available in the reference replicon sets.

**Fig. 3.**
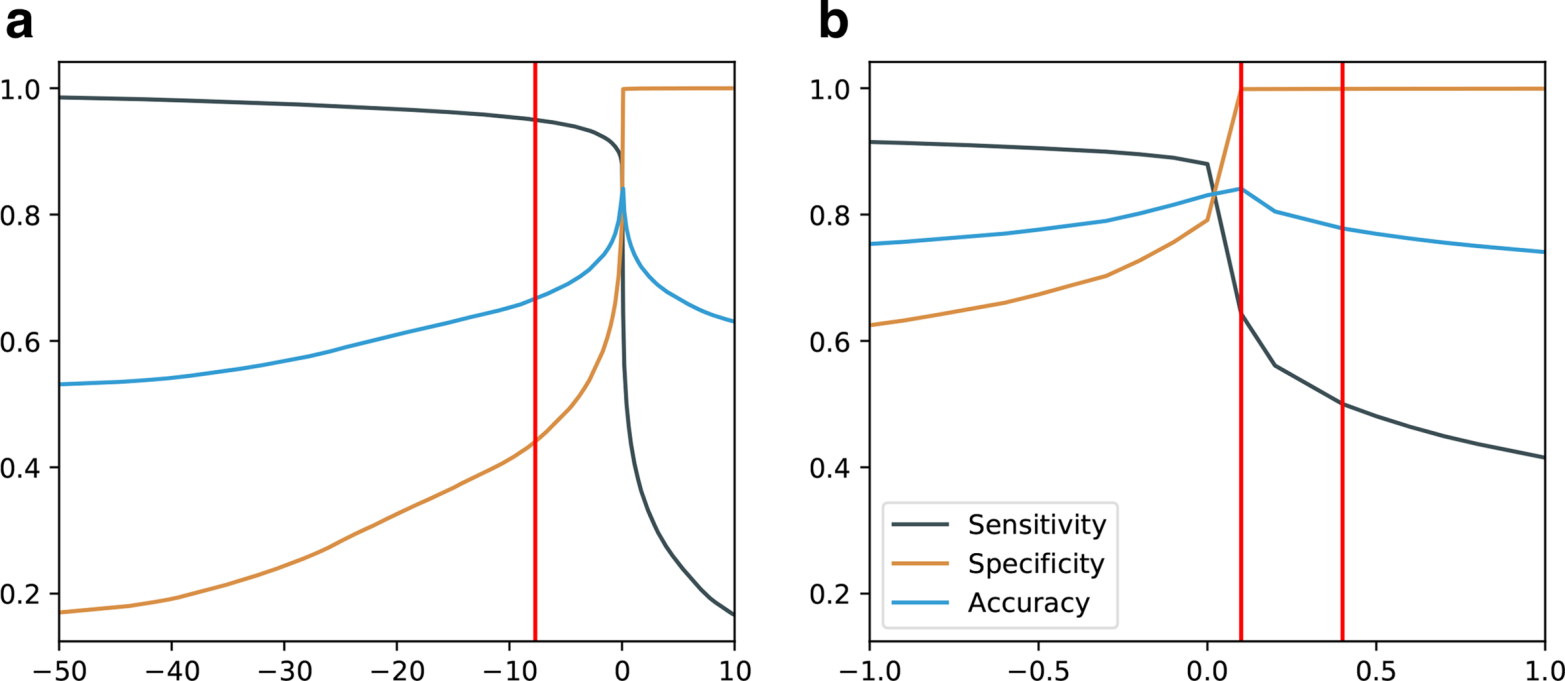
Evaluation statistics for replicon distribution score thresholds. Sensitivity, specificity and accuracy values are plotted against replicon distribution score threshold ranges. (a) Overview threshold range [−50,10]. (b) Detailed threshold range [−1,1]. Sensitivity is in black, specificity is in brown and accuracy is in blue. Red vertical lines from left to right: sensitivity threshold (−7.7), conservative threshold (0.1) and specificity threshold (0.4).

In order to assess the discriminative performance of RDS regarding the replicon origin of contigs, we tested a broad range of thresholds computing sensitivity, specificity and accuracy metrics. The sensitivity, specificity and accuracy values for a range of RDS thresholds are plotted in Fig. 3. The sensitivity and specificity curves follow a sharp inflection point near the default RDS value, i.e. 0. We attribute this behaviour to contigs harbouring protein sequences that are not covered by the MPS database. To overcome this limitation and achieve both sensitive and specific classifications, we defined three distinct thresholds: (i) an SNT; (ii) an SPT; (iii) a CT set to 95 % sensitivity, 99.9 % specificity and the highest accuracy, respectively. Thus, contigs with an RDS smaller than the SNT can be classified as chromosomal while still retaining 95 % of all plasmid contigs. Correspondingly, contigs with an RDS larger than the SPT can be classified as plasmid fragments achieving a specificity ≥99.9 %. To compute actual values for these thresholds, we conducted classifications of Monte Carlo replicon fragment simulations (*n*=1 564 639) by which the following values were established: SNT=−7.7, SPT=0.4 and CT=0.1 at a maximal accuracy of 84.1 %. These values surround the inflection point near 0 and were henceforth used as the final discrimination thresholds in the Platon implementation.

To finally assess the RDS-based contig classification, a comprehensive performance benchmark was conducted. To do this, we created simulated short reads based on all complete NCBI RefSeq genomes (*n*=13 930) covering a broad range of bacterial taxa. The resulting short reads were then reassembled into contigs (*n*=820 392), which were aligned back to the original genomes, thus creating our ground truth. This benchmark dataset comprised a total of 63 107 true plasmid contigs. All contigs were classified by their mean RDS value, applying the computed SNT and SPT thresholds. This RDS workflow classified 38 197 plasmid contigs and 754 082 chromosomal contigs correctly, thus achieving an accuracy of 0.966 and a sensitivity of 0.605, as well as an F1 score of 0.731 and an MCC of 0.732, calculated using the following confusion matrix: *tp*=38 197, *tn*=754 082, *fp*=3 203, *fn*=24 910.

Although the RDS approach achieved an accuracy of 0.966, it still misclassified 24 910 true plasmid contigs and 3 203 true chromosomal contigs. It is common knowledge that certain proteins are encoded on both replicon types, for instance, relaxases and type4-coupling proteins (T4CP) – key proteins of integrative conjugative elements [45]. To assess the discriminative power of the RDS metric on these widespread protein classes we extracted a set of 4 683 relaxase and 2 151 T4CP clusters from the MPS database by MOBscan [39] and TXSscan [46] HMM profile searches and investigated the range of related RDS values (Fig. S1); 73 and 66 % from the relaxase (*n*=3 321) and T4CP (*n*=1 436) protein clusters had an RDS between −0.5 and 0.5 and thus can be considered to be quite equally distributed. Small contigs solely or mainly encoding these protein sequences could therefore be especially hard to classify by the RDS metric. However, we also found 817 and 411 protein clusters that were quite chromosomally biased with a related RDS below −0.5 and extremes reaching values of −64.96 and −37.47 for the relaxases and T4CP, respectively. In addition, 445 and 304 protein clusters were quite plasmid biased with a related RDS above 0.5 and extremes reaching values of as high as 109.60 and 79.76 for the relaxases and T4CP, respectively. The latter protein clusters constitute approximately a quarter and a third of all relaxase and T4CP MPS subsets and have highly discriminative related RDS values. Hence, although there are protein classes harbouring many fairly equally distributed protein clusters, e.g. the analysed relaxases and T4CP, which are often encoded in very-hard-to-classify integrative conjugative elements, we still found MPSs with a strong predictive power regarding the replicon origin of a contig.

### Performance of the entire Platon workflow

As shown in the simulated short-read benchmark, the RDS metric achieved a high accuracy (ac=0.966) but rather moderate sensitivity (sn=0.605) due to the high number of false negatives (fn=24 910). In order to increase the detection rate of true plasmid contigs, the Platon workflow additionally comprises higher-level plasmid-related contig characterizations that serve as a basis for several heuristics. As both the protein homology search and the contig characterizations of large plasmids are computationally expensive, contigs >500 kbp are automatically assigned to the chromosome. To assess the potentially negative impact of this heuristic on the classification performance, contig length distributions for both replicon types within the simulated short-read dataset (Fig. S2) were investigated. In line with the smaller plasmid contig length on average, only 119 of 63 107 plasmid contigs were actually larger than 500 kbp compared to 15 750 of 757 285 chromosome contigs. Hence, only 0.19 % of all plasmid contigs were erroneously assigned to the chromosome, but 99.25 % of all contigs larger than 500 kbp were correctly classified by this heuristic, which thus qualifies as an eligible tradeoff between sensitivity and runtime.

To measure and assess the overall classification performance of the entire implemented workflow (Fig. 1), we conducted two benchmarks against contemporary command line tools: an untargeted benchmark against PlasFlow on the aforementioned simulated short-read dataset as well as a targeted benchmark against PlaScope and PlasmidFinder on sequenced *E. coli* isolates.

### Performance benchmark on taxonomically diverse simulated short-read assemblies

To assess the performance of the extended Platon workflow in an untargeted, i.e. taxon- independent, setup, we conducted a comprehensive benchmark against PlasFlow, a contemporary plasmid prediction tool for metagenomics that was presented to also be eligible for the recruitment of plasmid contigs from isolates. For this benchmark, all complete bacterial NCBI RefSeq genomes (*n*=13 930) covering a broad range of bacterial taxa were used to simulate short reads that were *de novo* assembled. The resulting contigs were then aligned back onto original genomes. A confusion matrix as well as common classifier performance metrics aggregated for all contigs (*n*=820 392) are shown in Table 1. In this benchmark Platon recruited 48 333 and PlasFlow 45 999 true plasmid contigs, resulting in comparable sensitivity and negative predictive values (NPV) of 0.762 and 0.729 and 0.98 and 0.975, respectively. However, PlasFlow predicted 17 times more false positives (fp=88 712) than Platon (fp=5 277). Due to the notably lower number of false positives, Platon clearly outperformed PlasFlow in terms of accuracy, specificity and positive predictive value (PPV), as well as the balanced metrics F1 score and Matthew’s correlation coefficient (MCC). An overview of how many contigs could be classified by which RDS threshold and heuristic filter is given in Table S3.

**Table 1.** Performance benchmark results computed contig-wise on simulated short-read data

Metric	PlasFlow	Platon
Accuracy	0.871	**0.976**
Sensitivity	0.729	**0.766**
Specificity	0.883	**0.993**
PPV	0.341	**0.902**
NPV	0.975	**0.981**
F1	0.465	**0.828**
MCC	0.440	**0.818**
TP	45 999	**48 333**
TN	668 573	**752 080**
FP	88 712	**5 277**
FN	17 108	**14 774**

**Fig. 4.**
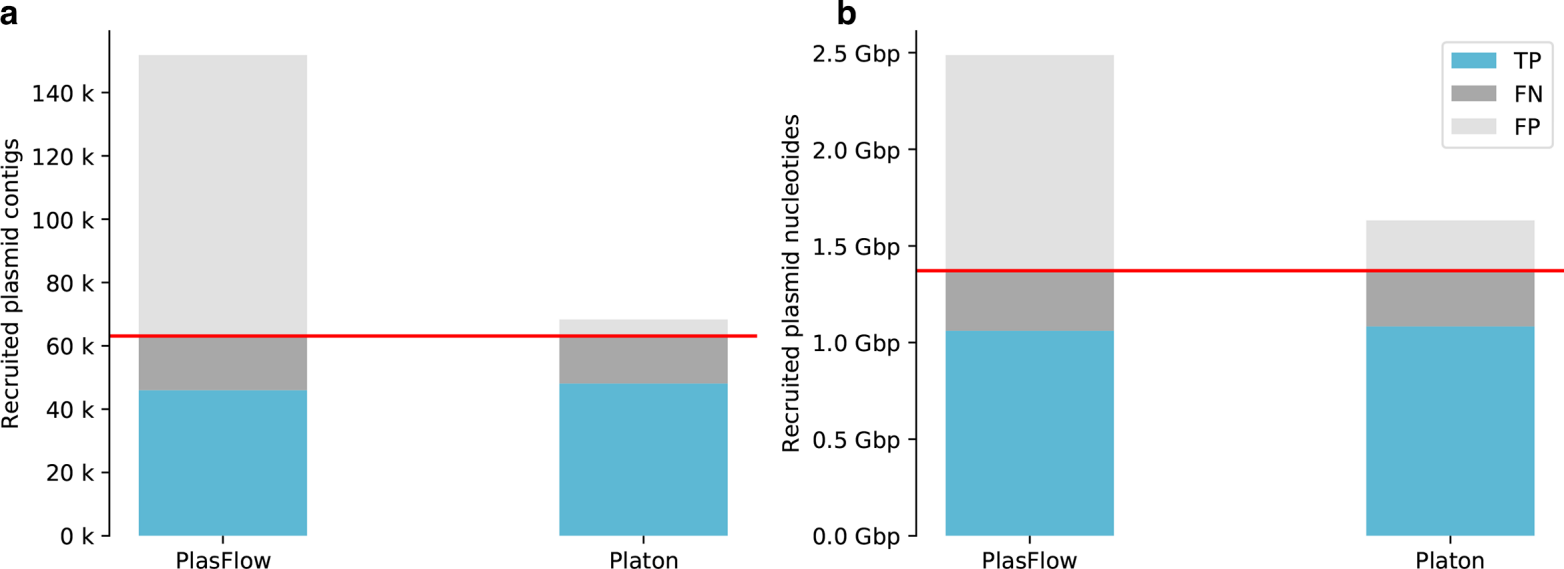
Performance benchmark metrics on simulated short-read data. A performance benchmark was conducted on all complete bacterial genomes of the NCBI RefSeq database, assembling simulated short reads and subsequently realigning them onto original genomes. For scaling reasons and the sake of readability, true negatives were discarded. (a) Benchmark results calculated contig-wise. Horizontal red line, total number of true plasmid contigs. (b) Benchmark results calculated nucleotide-wise. Horizontal red line, total number of true plasmid DNA nucleotides.

Due to different contig lengths, the mere number of correctly classified contigs might not always be congruent with the recruited plasmid content, which could play a vital role in downstream analyses, e.g. the recruitment of plasmid-borne genes or sequence motifs, such as oriT and oriV. Hence, benchmarks that only measure the number of classified contigs might, to some extent, be misleading, and so we complemented the former benchmark with a genomic content-based view calculating an additional confusion matrix based on classified DNA nucleotides (Table S4). Fig. 4 provides a combined view on both benchmark setups. In this complementary benchmark, the specificity values for PlasFlow increased from 0.883 contig-wise to 0.979 nucleotide-wise compared to stable and higher values for Platon (contig-wise=0.993; nucleotide-wise=0.995). The accuracy values also increased from 0.871 contig-wise to 0.974 nucleotide-wise for PlasFlow, whereas the accuracy values achieved by Platon only improved slightly (contig-wise=0.976; nucleotide-wise=0.99). Taking into account the genomic content of classified contigs revealed a performance improvement of PlasFlow in terms of accuracy and specificity, but it still fell slightly below Platon. However, PlasFlow predicted 4.3 times more false-positive plasmid nucleotides (fp=1 115.3 mbp) than Platon (fp=260.9 mbp), in line with the contig-wise benchmark.

**Fig. 5.**
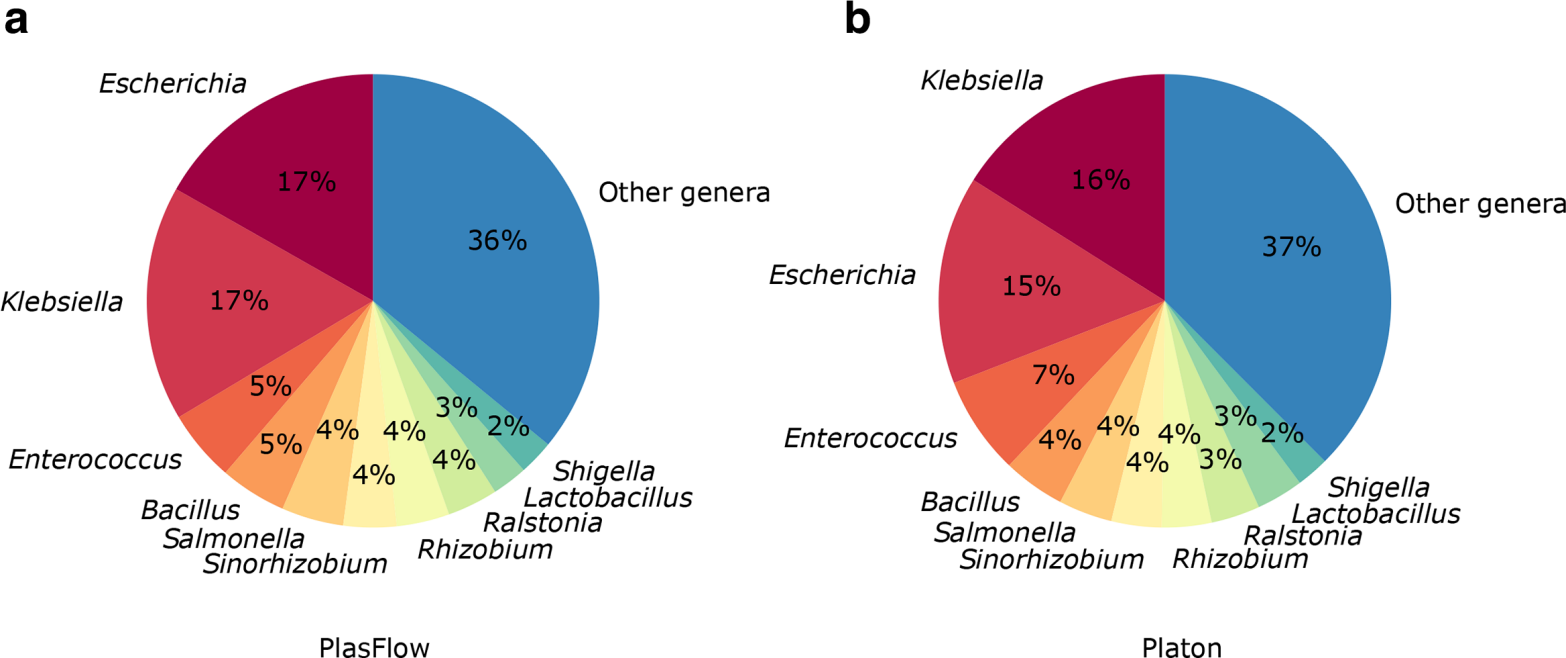
Taxonomic distribution of recruited plasmid contigs. The taxonomic distribution of the recruited plasmid contigs for the simulated benchmark dataset is shown binned to the genus level. Taxa accounting for less than 2 % are grouped as ‘others’. (a) PlasFlow; (b) Platon.

The taxonomic compositions of training datasets for machine learning approaches and prebuilt databases can have a severe impact on benchmark performance and the results of analyses. To assess a potential bias towards certain taxa we additionally analysed the taxonomic distribution of the recruited plasmid contigs of the simulated short-read dataset binned to the genus level (Fig. 5). The underlying benchmark dataset contained true plasmid contigs from 469 distinct genera and 1234 species. From these, Platon recruited plasmid contigs from 434 genera, whereas PlasFlow recruited plasmid contigs from 384 genera (Table S5). For both tools, the three taxa *Escherichia *, *Klebsiella* and *Enterococcus* accounted for nearly 40 % of the recruited sequences alike the taxonomic profile of the underlying benchmark dataset in which the aforementioned taxa accounted for 26 %. On a species level, Platon and PlasFlow recruited plasmid contigs from 1 128 and 1 014 distinct species, respectively, in line with the aforementioned genus-level results. Although PlasFlow was developed as an untargeted tool for metagenomics, Platon recruited plasmid contigs from a wider taxonomic range, thus demonstrating the competitive edge of the taxon-independent RDS approach complemented by contig characterization heuristics.

### Targeted performance benchmark on sequenced *E. coli* isolates

Simulated data seldom reflect the existing biological and technical complexity and the plethora of potential pitfalls. Hence, we additionally benchmarked the Platon workflow on real data in a targeted setup. We compared the performance of Platon against PlaScope and PlasmidFinder, which were both published as targeted approaches for the plasmid prediction within whole-genome sequencing data. PlaScope provides a precompiled *E. coli* database for download, which was used in this benchmark, and PlasmidFinder was specifically designed for the analysis of *Enterobacteriaceae* genomes. As the PlasmidFinder database is part of Platon’s contig characterization, we assessed its performance to transparently compare both tools side by side. For this benchmark the genomes of 24 *E. coli* isolates were sequenced using both Illumina short-read and Oxford Nanopore long-read technologies. For 21 isolates the hybrid assemblies resulted in closed chromosomes, which were used as the ground truth data. Contigs from short read-only assemblies (*n*=1 337) were aligned to the closed assemblies and used as the actual benchmark data. Table 2 shows the confusion matrix as well as computed benchmark metrics. PlasmidFinder achieved the lowest false-positive rate (fp=14) resulting in the highest specificity of 0.987, closely followed by Platon (*sp*=0.966) and PlaScope (*sp*=0.952), but showed the lowest true-positive rate (tp=57) and sensitivity (sn=0.223), thus performing worse than Platon (sn=0.699) and PlaScope (sn=0.684). With regard to accuracy, PPV, NPV, F1 score and MCC metrics, Platon and PlaScope performed nearly on par, although Platon was slightly ahead on each. Both tools performed better than PlasmidFinder on these metrics. This was especially true for the balanced metrics F1 score and MCC, for which Platon and Plascope clearly outperformed PlasmidFinder.

**Table 2.** Performance benchmark results contig-wise on sequenced isolate short-read data

Metric	PlaScope	PlasmidFinder	Platon
Accuracy	0.901	0.841	**0.915**
Sensitivity	0.684	0.223	**0.699**
Specificity	0.952	**0.987**	0.966
PPV	0.771	0.803	**0.829**
NPV	0.927	0.843	**0.931**
F1	0.725	0.349	**0.758**
MCC	0.666	0.368	**0.711**
TP	175	57	**179**
TN	1 029	**1 067**	1 044
FP	52	**14**	37
FN	81	199	**77**

**Fig. 6.**
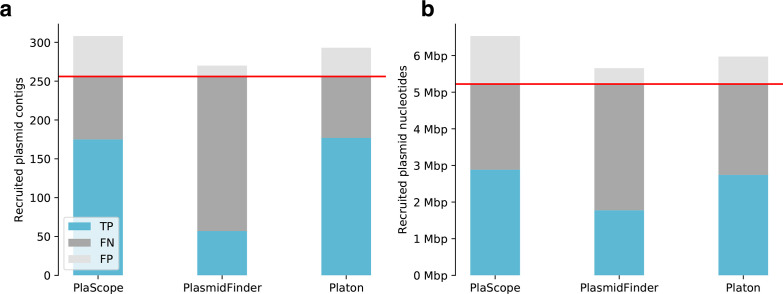
Performance benchmark metrics on real short-read data. A performance benchmark was conducted on 21 *E. coli* genomes, for which both short-read draft assemblies and complete genomes via hybrid assemblies were available. For scaling reasons and the sake of readability, true negatives were discarded. (a) Benchmark results calculated contig-wise. Horizontal red line, total number of true plasmid contigs. (b) Benchmark results calculated nucleotide-wise. Horizontal red line, total number of true plasmid DNA nucleotides.

Similarly, with the simulated short-read benchmark we also compared the performances of Platon, PlaScope and PlasmidFinder, taking into account the amount of genomic content (Fig. 6) computed on a nucleotide-wise confusion matrix (Table S6). The nucleotide-wise results were in line with those calculated contig-wise: PlasmidFinder had the lowest number of false positives, but also detected remarkably fewer plasmid nucleotides than PlaScope and Platon. The latter two detected a nearly equal quantity of plasmid content, with Platon predicting notably fewer false positives than PlaScope.

### Conclusion

Due to the complex nature of plasmid fragments, replicon type classification, i.e. prediction of origin, for contigs resulting from short-read draft assemblies is a difficult task. Many different methods and tools have recently been described in the literature, but few work on draft assemblies only, are implemented in a high-throughput savvy manner or provide statistically balanced predictions in an untargeted, i.e. taxon-independent manner.

To tackle this issue, we investigated the natural distribution biases of protein-coding genes between chromosomes and plasmids for a large set of protein sequences in bacteria. In this study, we defined, computed and tested statistical discrimination thresholds for the introduced new metric RDS and showed that it is a feasible approach to the problem. However, small contigs without sufficient protein sequences or contigs encoding for protein sequences that were either not covered by the MPS database or equally distributed between chromosomes and plasmids remained hard to classify correctly. However, even for the protein classes relaxases and T4CP, which are often found on notoriously hard-to-classify integrative conjugative elements, we found protein sequences with strong predictive power. To mitigate these drawbacks and improve the overall sensitivity, we complemented this approach with several heuristics exploiting higher-level plasmid-related sequence characterizations. We implemented this new workflow in a software tool called Platon and conducted benchmarks against three contemporary software tools, i.e. PlaScope, PlasFlow and PlasmidFinder on both simulated short-read data and sequenced isolates.

Analysing a large set of diverse bacterial species, Platon achieved equal sensitivity but higher accuracy and specificity than PlasFlow, while the predictions made by Platon were more balanced in terms of F1 score and MCC due to a low number of false positives.

Even though the underlying MPS database follows an untargeted approach, i.e. it is not restricted to or focused on certain taxa, Platon achieved competitive results compared to the targeted tools PlaScope and PlasmidFinder in a benchmark using real sequencing data for *E. coli* isolates. In both benchmarks Platon achieved the highest sensitivity and accuracy, thus endorsing the exploitation of the natural replicon distribution biases of protein-coding genes as an eligible method for the large-scale, high-throughput, taxon-independent prediction of plasmid-borne contigs from short-read draft assemblies.

Implemented as a multithreaded, locally executable Linux command line application in Python 3, we also envision it as an appropriate fit for integration into larger analysis pipelines as well as an upfront tool for subsequent plasmid-specific analyses. For the sake of a streamlined integration and installation, all necessary third party executables are bundled with the software. All source code and documentation are freely available under a GPL3 license and hosted at GitHub (https://github.com/oschwengers/platon) and http://platon.computational.bio/. For further convenience, Platon is also available as a BioConda package (platon) and via PyPI (cb-platon). A prebuilt database is hosted at Zenodo (DOI: 10.5281/zenodo.3349652).

Future developments will include the addition of new higher-level contig characterizations as well as further enhancements of applied heuristics.

## Data Bibliography

1. Platon was developed as a Python 3 command line application for Linux.

2. The complete source code and documentation are available on GitHub under a GPL3 license: https://github.com/oschwengers/platon and http://platon.computational.bio.

3. All database versions are hosted at Zenodo (DOI: 10.5281/zenodo.3349651).

4. Platon is available via the bioconda package platon.

5. Platon is available via the PyPI package cb-platon.

6. The bacterial representative sequences for UniProt’s UniRef90 protein clusters, complete bacterial genome sequences from the NCBI RefSeq database, complete plasmid sequences from the NCBI genomes plasmid section, created artificial contigs, RDS threshold metrics and raw protein replicon hit counts used to create and evaluate the marker protein sequence database are hosted at Zenodo (DOI: 10.5281/zenodo.37591697). Twenty-four *Escherichia coli* isolates sequenced with short-read (Illumina MiSeq) and long-read sequencing technologies (Oxford Nanopore Technology GridION platform) used for real data benchmarks are available under the following NCBI BioProjects: PRJNA505407 and PRJNA387731.

## Supplementary Data

Supplementary material 1
