# Supplementary material for: Platon: identification and characterization of bacterial plasmid contigs in short-read draft assemblies exploiting protein sequence-based replicon distribution scores
Source: Microb Genom. 2020 Jun 24;6(10):mgen000398. doi: 10.1099/mgen.0.000398 (PMC7660248; doi:10.1099/mgen.0.000398)
Supplement: Supplementary material 1 [file mgen-6-398-s001.pdf]

Supplementary Table S1. Regular expressions used for PCLA cluster extraction for subsequent HMM creation

Type	Regular Expression
conjugation	Tra[^ICG]<br>Trb[A-Z]<br>Trw[^ABC]<br>VirB[0-9]
replication	RepH<br>SopA<br>KorB<br>ParM<br>ParR<br>.*(plasmid).+(partition).*<br>.*(plasmid).+(rep)

Supplementary Table S2. Isolated and sequenced *Escherichia coli* genomes used in the real data benchmark.

Isolate	SRA Accession ID	Host	Assembled Contigs from Short Reads >= 1 kbp	Chromosome Closed in Hybrid Assemblies	Closed Plasmids in Hybrid Assemblies
H69	SRX5007771 SRX5007759	Homo sapiens	69	yes	2
H100	SRX5007774 SRX5007760	Homo sapiens	44	yes	3
H105	SRX5002893 SRX5002892	Homo sapiens	59	yes	4
H108	SRX5007773 SRX5007761	Homo sapiens	69	yes	3
H113	SRX5007776 SRX5007762	Homo sapiens	35	yes	2
H136	SRX5007775 SRX5007763	Homo sapiens	77	yes	5
H157	SRX5007770 SRX5007756	Homo sapiens	87	yes	4
H162	SRX5007769 SRX5007757	Homo sapiens	56	yes	1
H176	SRX5007772 SRX5007758	Homo sapiens	92	yes	2
V1	SRX5007768 SRX5007764	Canis lupus	61	yes	6
V8	SRX5007790 SRX5007782	Equus caballus	46	yes	2
V9	SRX5007786 SRX5007784	Equus caballus	118	yes	5
V41	SRX5007794 SRX5007777	Canis lupus	82	yes	3
V64	SRX5007789 SRX5007780	Canis lupus	52	yes	2
V71	SRX5007788 SRX5007766	Canis lupus	90	yes	7
V73	SRX5007791 SRX5007783	Equus caballus	65	yes	2
V79	SRX6897800	Equus caballus	55	no	5

	SRX6897801				
V80	SRX5007787<br>SRX5007785	Equus caballus	68	yes	0
V173	SRX5007767<br>SRX5007765	Equus caballus	75	yes	1
V177	SRX5007793<br>SRX5007781	Canis lupus	66	yes	3
V195	SRX5007792<br>SRX5007779	Canis lupus	51	yes	4
V292	SRX5007795<br>SRX5007778	Canis lupus	99	yes	3
V215	SRX6897802<br>SRX6897803	Equus caballus		no	6
V232	SRX6897804<br>SRX6897805	Canis lupus		no	1

Supplementary Table S3. Number of classified contigs for each RDS and length threshold and characterization heuristic implemented in the Platon workflow for both simulated and real benchmarks.

RDS threshold / heuristic	Simulated data	Real data
Length < 1kb	0	0
Length >= 500 kb	15,869	22
RDS SNT	443,159	985
RDS SPT	42,669	241
RDS CT	50,525	212
Circularity	53,611	266
Incompatibility group	5,749	82
Replication gene	6,772	48
Mobilization gene	287	0
OriT	1,614	40

Supplementary Table S4. Confusion matrix for the untargeted simulated short-read data benchmark computed by classified genomic content measured in contig nucleotides.

Metric	PlasFlow	Platon
TP	1,061,149,767	1,087,412,371
TN	51,894,599,885	52,749,014,593
FP	1,115,299,457	260,884,749
FN	310,703,527	284,440,923

Supplementary Table S5. Taxa of bacterial genomes for which true plasmid contigs have been correctly identified by each tool in the simulated short-read benchmark binned to the *genus* taxon. Aggregated counts for each *genus* are provided in parenthesis.

PlasFlow	Platon
Escherichia (7585)	*Klebsiella* (7651)
Klebsiella (7577)	*Escherichia* (7099)
Enterococcus (2309)	*Enterococcus* (3382)
Bacillus (2150)	*Bacillus* (2109)
Salmonella (2002)	*Salmonella* (1835)
Sinorhizobium (1713)	*Sinorhizobium* (1706)
Rhizobium (1707)	*Rhizobium* (1684)
Ralstonia (1633)	*Ralstonia* (1659)
Lactobacillus (1139)	*Lactobacillus* (1596)
Shigella (1124)	*Shigella* (1109)
Enterobacter (898)	*Enterobacter* (881)
Xanthomonas (653)	*Acinetobacter* (792)
Acinetobacter (647)	*Piscirickettsia* (750)
Staphylococcus (645)	*Acetobacter* (728)
Acetobacter (591)	*Xanthomonas* (641)
Citrobacter (570)	*Staphylococcus* (638)
Pseudomonas (534)	*Citrobacter* (571)
Azospirillum (504)	*Pseudomonas* (539)
Piscirickettsia (489)	*Borreliella* (503)
Yersinia (456)	*Yersinia* (464)
Burkholderia (433)	*Phaeobacter* (429)
Borreliella (336)	*Azospirillum* (418)
Vibrio (326)	*Borrelia* (380)
Sphingobium (319)	*Burkholderia* (363)
Rhodococcus (296)	*Vibrio* (339)
Borrelia (292)	*Sphingobium* (310)
Lactococcus (285)	*Lactococcus* (308)
Phaeobacter (283)	*Rhodococcus* (258)
Microvirga (260)	*Streptomyces* (258)
Agrobacterium (259)	*Paracoccus* (249)
Paracoccus (234)	*Agrobacterium* (242)
Paraburkholderia (232)	*Deinococcus* (216)
Streptomyces (208)	*Aeromonas* (200)
Aeromonas (183)	*Microvirga* (165)
Deinococcus (173)	*Clostridium* (163)
Nostoc (162)	*Nostoc* (153)
Cupriavidus (161)	*Mycobacterium* (145)
Sphingomonas (157)	*Cupriavidus* (145)
Rhodobacter (151)	*Pantoea* (139)
Mycobacterium (151)	*Pediococcus* (135)
Pantoea (143)	*Pseudonocardia* (128)
Methylobacterium (142)	*Campylobacter* (121)
Novosphingobium (128)	*Novosphingobium* (120)
Komagataeibacter (123)	*Thermus* (118)
Bradyrhizobium (114)	*Komagataeibacter* (118)
Leclercia (112)	*Sphingomonas* (114)
Shewanella (103)	*Rhodobacter* (113)
Raoultella (101)	*Paraburkholderia* (108)

Pediococcus (99)	*Raoultella* (106)
Ensifer (96)	*Moraxella* (102)
Pandoraea (91)	*Shewanella* (102)
Clostridium (89)	*Leuconostoc* (96)
Pseudonocardia (85)	*Ensifer* (96)
Thermus (82)	*Sulfitobacter* (95)
Sulfitobacter (80)	*Leclercia* (92)
Acaryochloris (75)	*Methylobacterium* (91)
Ochrobactrum (69)	*Bradyrhizobium* (91)
Campylobacter (68)	Listeria (82)
Acidiphilium (67)	*Arsenophonus* (79)
Serratia (66)	*Legionella* (77)
Mesorhizobium (62)	*Gloeothece* (77)
Gloeothece (62)	*Acaryochloris* (76)
Roseomonas (61)	*Pandoraea* (73)
Photobacterium (60)	*Candidatus* (71)
Arsenophonus (59)	*Sphingopyxis* (67)
Methylorubrum (57)	*Synechococcus* (65)
Sphingopyxis (57)	*Paenibacillus* (62)
Kozakia (55)	*Ochrobactrum* (60)
Arthrobacter (54)	*Arthrobacter* (60)
Leptolyngbya (51)	*Chlamydia* (59)
Leuconostoc (50)	*Photobacterium* (59)
Enterobacteriaceae (49)	*Serratia* (58)
Shinella (48)	*Acidiphilium* (57)
Phytobacter (48)	*Synechocystis* (57)
Listeria (47)	*Leptolyngbya* (56)
Proteus (44)	*Azotobacter* (51)
Cronobacter (43)	*Kozakia* (51)
Candidatus (40)	*Cronobacter* (50)
Gluconobacter (39)	*Zymomonas* (48)
Mycoplasma (39)	*Enterobacteriaceae* (47)
Synechococcus (38)	*Aminobacter* (46)
Moraxella (37)	*Phytobacter* (46)
Synechocystis (36)	*Proteus* (43)
Haematobacter (36)	*Methylorubrum* (42)
Helicobacter (35)	*Clavibacter* (41)
Aminobacter (33)	*Shinella* (41)
Paenibacillus (32)	*Mesorhizobium* (40)
Acidovorax (32)	*Acidovorax* (37)
Corynebacterium (31)	*Roseomonas* (37)
Mycolicibacterium (31)	*Geobacillus* (36)
Streptococcus (30)	*Corynebacterium* (36)
Neorhizobium (29)	*Gluconobacter* (35)
Carnobacterium (29)	*Neisseria* (34)
Croceicoccus (29)	*Leptospira* (33)
Azotobacter (28)	*Weissella* (31)
Geobacillus (28)	*Acidithiobacillus* (31)
Erwinia (26)	*Rickettsia* (31)
Celeribacter (25)	*Helicobacter* (30)
Antarctobacter (24)	*Haematobacter* (30)
Legionella (23)	*Psychrobacter* (29)
Aliivibrio (23)	*Meiothermus* (29)
Oscillatoria (23)	*Leisingera* (29)
Chlamydia (22)	*Celeribacter* (29)
Xylella (22)	*Planococcus* (28)

Stanieria (22)	*Calothrix* (28)
Aureimonas (22)	*Stanieria* (28)
Confluentimicrobium (22)	*Polaromonas* (28)
Phyllobacterium (22)	*Erwinia* (27)
Zymomonas (21)	*Carnobacterium* (27)
Xenorhabdus (21)	*Oscillatoria* (27)
Nitrobacter (21)	*Streptococcus* (26)
Rahnella (20)	*Pseudanabaena* (26)
Rhodovulum (20)	*Xylella* (25)
Octadecabacter (20)	*Antarctobacter* (25)
Aromatoleum (20)	*Yangia* (25)
Rhizorhabdus (20)	*Indioceanicola* (25)
Neisseria (19)	*Rhodovulum* (24)
Gordonia (19)	*Croceicoccus* (24)
Yangia (19)	*Neorhizobium* (23)
Epibacterium (19)	*Mycolicibacterium* (23)
Buchnera (18)	*Martelella* (23)
Martelella (18)	*Cyanothece* (23)
Indioceanicola (18)	*Bacteroides* (22)
Rippkaea (18)	*Ruminococcus* (22)
Leptospira (17)	*Rippkaea* (22)
Weissella (17)	*Buchnera* (21)
Nocardia (17)	*Aliivibrio* (21)
Psychrobacter (17)	*Anabaena* (21)
Thioclava (17)	*Granulicella* (20)
Edwardsiella (16)	*Confluentimicrobium* (20)
Anabaena (16)	*Pseudoalteromonas* (19)
Cyanothece (16)	*Marinovum* (19)
Alteromonas (15)	*Deferribacter* (19)
Rickettsia (15)	*Phyllobacterium* (19)
Polaromonas (15)	*Methylosinus* (18)
Sagittula (15)	*Ilyobacter* (18)
Methylosinus (14)	*Aromatoleum* (18)
Planococcus (14)	*Salipiger* (18)
Marinobacter (14)	*Aureimonas* (18)
Calothrix (14)	*Geminocystis* (18)
Leisingera (14)	*Peptoclostridium* (17)
Dinoroseobacter (14)	*Ketogulonicigenium* (17)
Salipiger (14)	*Epibacterium* (17)
Pelagibaca (14)	*Pelagibaca* (17)
Defluviimonas (14)	*Xenorhabdus* (16)
Spiroplasma (13)	*Desulfovibrio* (16)
Clavibacter (13)	*Rhizorhabdus* (16)
Microcoleus (13)	*Sedimentitalea* (16)
Sedimentitalea (13)	*Edwardsiella* (15)
Metakosakonia (13)	*Rahnella* (15)
Marinovum (12)	*Pseudarthrobacter* (15)
Pseudanabaena (12)	*Sagittula* (15)
Alicycliphilus (12)	*Pseudorhodobacter* (15)
Gemmobacter (12)	*Alteromonas* (14)
Francisella (11)	*Nocardia* (14)
Acidithiobacillus (11)	*Salinibacter* (14)
Oligotropha (11)	*Gemmatirosa* (14)
Macrococcus (11)	*Gemmobacter* (14)
Kosakonia (11)	*Metakosakonia* (14)
Crinalium (11)	*Fusobacterium* (13)

Trichormus (11)	*Nitrobacter* (13)
Chelativorans (11)	*Selenomonas* (13)
Bosea (11)	*Lysinibacillus* (13)
Geminocystis (11)	*Dinoroseobacter* (13)
Frondihabitans (11)	*Methylomonas* (13)
Pseudoalteromonas (10)	*Microcoleus* (13)
Pseudarthrobacter (10)	*Francisella* (12)
Chondrocystis (10)	*Coxiella* (12)
Buttiauxella (10)	*Parageobacillus* (12)
Pseudorhodobacter (10)	*Mycoplasma* (12)
Lysinibacillus (9)	*Marinobacter* (12)
Ruegeria (9)	*Octadecabacter* (12)
Tistrella (9)	*Ruegeria* (12)
Yoonia (9)	*Methylocystis* (12)
Massilia (9)	*Crinalium* (12)
Hymenobacter (9)	*Defluviimonas* (12)
Niveispirillum (9)	*Acidisarcina* (12)
Acidisarcina (9)	*Nitrosomonas* (11)
Bifidobacterium (8)	*Rubrobacter* (11)
Mycobacteroides (8)	*Asticcacaulis* (11)
Meiothermus (8)	*Chondrocystis* (11)
Ketogulonicigenium (8)	*Treponema* (10)
Dietzia (8)	*Chelativorans* (10)
Cedecea (8)	*Phenylobacterium* (10)
Deferribacter (8)	*Halomonas* (10)
Granulicella (8)	*Thioclava* (10)
Morganella (7)	*Citricoccus* (10)
Bacteroides (7)	*Xanthobacter* (9)
Virgibacillus (7)	*Bifidobacterium* (9)
Tetragenococcus (7)	*Achromobacter* (9)
Methylibium (7)	*Methylibium* (9)
Methylocystis (7)	*Sulfuricurvum* (9)
Cryobacterium (7)	*Alicycliphilus* (9)
Blastomonas (7)	*Yoonia* (9)
Xanthobacter (6)	*Kosakonia* (9)
Pectobacterium (6)	*Massilia* (9)
Providencia (6)	*Hymenobacter* (9)
Ruminococcus (6)	*Bosea* (9)
Parageobacillus (6)	*Niveispirillum* (9)
Anoxybacillus (6)	*Hoeflea* (9)
Paenarthrobacter (6)	*Planctomyces* (9)
Rhodoferax (6)	*Providencia* (8)
Kocuria (6)	*Clostridioides* (8)
Halomonas (6)	*Gordonia* (8)
Rhizobiales (6)	Haliscomenobacter (8)
Porphyrobacter (6)	*Roseobacter* (8)
Citricoccus (6)	*Oligotropha* (8)
Coxiella (5)	*Pannonibacter* (8)
Clostridioides (5)	*Tistrella* (8)
Roseobacter (5)	*Frondihabitans* (8)
Thauera (5)	*Runella* (8)
Achromobacter (5)	*Buttiauxella* (8)
Methylocella (5)	*Pectobacterium* (7)
Aster (5)	*Morganella* (7)
Hoeflea (5)	*Rhodothermus* (7)
Microbacterium (5)	*Melissococcus* (7)

Rhodobacteraceae (5)	*Myroides* (7)
Acidibrevibacterium (5)	*Chryseobacterium* (7)
Tabrizicola (5)	*Dietzia* (7)
Crocosphaera (5)	*Trichormus* (7)
Beijerinckia (4)	*Kocuria* (7)
Plesiomonas (4)	*Acidibrevibacterium* (7)
Actinobacillus (4)	*Virgibacillus* (6)
Pasteurella (4)	*Desulfobacterium* (6)
Nitrosomonas (4)	*Arcobacter* (6)
Thiomonas (4)	*Lawsonia* (6)
Selenomonas (4)	*Paenarthrobacter* (6)
Allochromatium (4)	*Tetragenococcus* (6)
Microcystis (4)	*Sodalis* (6)
Brevibacillus (4)	*Caulobacter* (6)
Kitasatospora (4)	*Macrococcus* (6)
Tsukamurella (4)	*Rhodoferax* (6)
Lawsonia (4)	*Simkania* (6)
Gluconacetobacter (4)	*Amycolatopsis* (6)
Glutamicibacter (4)	*Cedecea* (6)
Nodularia (4)	*Thalassospira* (6)
Myroides (4)	*Methylocella* (6)
Asticcacaulis (4)	*Singulisphaera* (6)
Desulfovibrio (4)	*Aquabacterium* (6)
Labrenzia (4)	*Rhodobacteraceae* (6)
Phenylobacterium (4)	*Clostridiaceae* (6)
Exiguobacterium (4)	*Lelliottia* (6)
Oscillibacter (4)	*Tabrizicola* (6)
Vagococcus (4)	*Plesiomonas* (5)
Neokomagataea (4)	*Nitrosococcus* (5)
Plautia (4)	*Nitrosospira* (5)
Swingsia (4)	*Pelobacter* (5)
Hartmannibacter (4)	*Mycobacteroides* (5)
Clostridiaceae (4)	*Butyrivibrio* (5)
Simplicispira (4)	*Chroococcidiopsis* (5)
Bordetella (3)	*Labrenzia* (5)
Hafnia (3)	*Roseovarius* (5)
Fusobacterium (3)	*Tateyamaria* (5)
Rhodospirillum (3)	*Exiguobacterium* (5)
Halobacillus (3)	*Oscillibacter* (5)
Desulfobacterium (3)	*Flammeovirga* (5)
Bartonella (3)	*Thermaerobacter* (5)
Sodalis (3)	*Porphyrobacter* (5)
Pannonibacter (3)	*Brachyspira* (4)
Salinibacter (3)	*Hydrogenophilus* (4)
Roseovarius (3)	*Thiomonas* (4)
Thalassospira (3)	*Rhodospirillum* (4)
Alicyclobacillus (3)	*Halobacillus* (4)
Chelatococcus (3)	*Prevotella* (4)
Salimicrobium (3)	*Geobacter* (4)
Haematospirillum (3)	*Glutamicibacter* (4)
Erythrobacter (3)	*Thauera* (4)
Nostocales (3)	*Curtobacterium* (4)
Glaesserella (3)	*Thioflavicoccus* (4)
Lelliottia (3)	*Bartonella* (4)
Thiomicrorhabdus (3)	*Blattabacterium* (4)
Hydrocarboniclastica (3)	*Geoalkalibacter* (4)

Vitreoscilla (2)	*Neokomagataea* (4)
Hydrogenophilus (2)	*Cryobacterium* (4)
Sebaldella (2)	*Azoarcus* (4)
Finegoldia (2)	*Gloeocapsa* (4)
Cutibacterium (2)	*Haematospirillum* (4)
Haliscomenobacter (2)	*Blastomonas* (4)
Brochothrix (2)	*Cnuibacter* (4)
Pelobacter (2)	*Microbacterium* (4)
Caldicellulosiruptor (2)	*Actinobacillus* (3)
Desulfohalobium (2)	*Allochromatium* (3)
Prevotella (2)	*Microcystis* (3)
Chroococcidiopsis (2)	*Brevibacillus* (3)
Thioflavicoccus (2)	*Tsukamurella* (3)
Moritella (2)	*Spiroplasma* (3)
Simkania (2)	*Caldicellulosiruptor* (3)
endosymbiont (2)	*Desulfohalobium* (3)
Caulobacter (2)	*Nodularia* (3)
Rivularia (2)	*Aster* (3)
Cyanobacterium (2)	*Thermovirga* (3)
Singulisphaera (2)	*Thermobacillus* (3)
Hoyosella (2)	*Cyanobacterium* (3)
Azoarcus (2)	*Vagococcus* (3)
Polymorphum (2)	*Salimicrobium* (3)
Dickeya (2)	*Neochlamydia* (3)
Gloeocapsa (2)	*Simplicispira* (3)
Aquabacterium (2)	*Chromobacterium* (3)
Halocynthiibacter (2)	*Thiomicrorhabdus* (3)
Euzebya (2)	*Silvanigrellales* (3)
Cnuibacter (2)	*Crocosphaera* (3)
Planctomyces (2)	*Vitreoscilla* (2)
Nitratireductor (2)	*Bordetella* (2)
Brachybacterium (2)	*Beijerinckia* (2)
Gammaproteobacteria (2)	*Hafnia* (2)
Sterolibacteriaceae (2)	*Pasteurella* (2)
Thermaerobacter (2)	*Sebaldella* (2)
Comamonas (1)	*Finegoldia* (2)
Alcaligenes (1)	*Dermacoccus* (2)
Histophilus (1)	*Streptosporangium* (2)
Gallibacterium (1)	*Gluconacetobacter* (2)
Marivirga (1)	*Streptobacillus* (2)
Rhodopseudomonas (1)	*Sinomonas* (2)
Prosthecochloris (1)	*Tatumella* (2)
Nitrosospira (1)	*Pseudodesulfovibrio* (2)
Dermacoccus (1)	*Desulfocapsa* (2)
Brevibacterium (1)	*endosymbiont* (2)
Peptoclostridium (1)	*Cardinium* (2)
Acidipropionibacterium (1)	*Methylovorus* (2)
Desulfurella (1)	*Jannaschia* (2)
Zymobacter (1)	*Anoxybacillus* (2)
Sinomonas (1)	*Advenella* (2)
Rubrobacter (1)	*Rivularia* (2)
Hydrogenophaga (1)	*Natranaerobius* (2)
Wigglesworthia (1)	*Thioalkalivibrio* (2)
Tatumella (1)	*Maritalea* (2)
Waddlia (1)	*Calditerrivibrio* (2)
Mannheimia (1)	*Rufibacter* (2)

Solibacillus (1)	*Opitutaceae* (2)
Brachyspira (1)	*Desulfosporosinus* (2)
Desulfotalea (1)	*Plautia* (2)
Halobacteriovorax (1)	*Halioglobus* (2)
Kineococcus (1)	*Polymorphum* (2)
Rummeliibacillus (1)	*Dickeya* (2)
Cardinium (1)	*Altererythrobacter* (2)
Methylovorus (1)	*Capnocytophaga* (2)
Jannaschia (1)	*Paludisphaera* (2)
Photorhabdus (1)	*Cetia* (2)
Tateyamaria (1)	*Hartmannibacter* (2)
Advenella (1)	*Rickettsiales* (2)
Geobacter (1)	*Erythrobacter* (2)
Verminephrobacter (1)	*Nostocales* (2)
Natranaerobius (1)	*Thalassococcus* (2)
Thioalkalivibrio (1)	*Glaesserella* (2)
Tessaracoccus (1)	*Gammaproteobacteria* (2)
Nitrosococcus (1)	*Catenovulum* (2)
Prauserella (1)	*Humibacter* (2)
Allofrancisella (1)	*Planctopirus* (1)
Frankia (1)	*Isosphaera* (1)
Jeotgalibaca (1)	*Comamonas* (1)
Methylophaga (1)	*Alcaligenes* (1)
Arcobacter (1)	*Histophilus* (1)
Pusillimonas (1)	*Gallibacterium* (1)
Moorea (1)	*Herbaspirillum* (1)
Catharanthus (1)	*Marivirga* (1)
Mucilaginibacter (1)	*Saprospira* (1)
Cycloclasticus (1)	*Prosthecochloris* (1)
Paludisphaera (1)	*Gottschalkia* (1)
Geosporobacter (1)	*Kitasatospora* (1)
Sedimenticola (1)	*Hirschia* (1)
Aquitalea (1)	*Brochothrix* (1)
Psychromicrobium (1)	*Turneriella* (1)
Spongiibacter (1)	*Desulfurella* (1)
Magnetospirillum (1)	*Zymobacter* (1)
Agarilytica (1)	*Eubacterium* (1)
Fischerella (1)	*Rothia* (1)
Paraphotobacterium (1)	*Hydrogenophaga* (1)
Sphingosinicella (1)	*Wigglesworthia* (1)
Amycolatopsis (1)	*Flavobacterium* (1)
Tenericutes (1)	*Waddlia* (1)
Marivivens (1)	*Mannheimia* (1)
Sporosarcina (1)	*Moritella* (1)
Sulfuriferula (1)	*Desulfotalea* (1)
Thalassococcus (1)	*Halobacteriovorax* (1)
Ahniella (1)	*Kineococcus* (1)
Mycetocola (1)	*Marinitoga* (1)
Butyricimonas (1)	*Carboxydocella* (1)
Miniimonas (1)	*Xylanimonas* (1)
Catenovulum (1)	*Thermovibrio* (1)
Runella (1)	*Methylomicrobium* (1)
Humibacter (1)	*Collimonas* (1)
Flammeovirga (1)	*Photorhabdus* (1)
Xylanibacterium (1)	*Persephonella* (1)
Xanthomonadaceae (1)	*Pontibacter* (1)

Rhodopseudomonas (1)	*Verminephrobacter* (1)
Tatumella (1)	*Alicyclobacillus* (1)
Nitrosospira (1)	*Oceanimonas* (1)
Fischerella (1)	*Prauserella* (1)
Mannheimia (1)	*Pelagibacterium* (1)
Photorhabdus (1)	*Phycisphaera* (1)
Spongiibacter (1)	*Allofrancisella* (1)
Jannaschia (1)	*Hoyosella* (1)
Methylovorus (1)	*Sulfuricella* (1)
Thalassococcus (1)	*Frankia* (1)
Alcaligenes (1)	*Jeotgalibaca* (1)
Catenovulum (1)	*Verrucosispora* (1)
Zymobacter (1)	*Pusillimonas* (1)
Tenericutes (1)	*Thiolapillus* (1)
Xanthomonadaceae (1)	*Elizabethkingia* (1)
Mucilaginibacter (1)	*Mesotoga* (1)
Jeotgalibaca (1)	*Catharanthus* (1)
Mycetocola (1)	*Magnetospira* (1)
Psychromicrobium (1)	*Swingsia* (1)
Hydrogenophaga (1)	*Mucilaginibacter* (1)
	Cycloclasticus (1)
	Serpentinomonas (1)
	Erysipelothrix (1)
	Sedimenticola (1)
	Halocynthiibacter (1)
	Aquitalea (1)
	Psychromicrobium (1)
	Spongiibacter (1)
	Mitsuaria (1)
	Magnetospirillum (1)
	Chelatococcus (1)
	Agarilytica (1)
	Fischerella (1)
	Paraphotobacterium (1)
	Nitratireductor (1)
	Sphingosinicella (1)
	Acetobacteraceae (1)
	Marivivens (1)
	Sporosarcina (1)
	Sulfuriferula (1)
	Brachybacterium (1)
	Ahniella (1)
	Sphingorhabdus (1)
	Butyricimonas (1)
	Sterolibacteriaceae (1)
	Hydrocarboniclastica (1)
	Oenococcus (1)
	Thermoactinomycetaceae (1)
	Streptomonospora (1)
	Rhizobiales (1)

Supplementary Table S6. Confusion matrix for the targeted real short-read/long-read hybrid data benchmark computed by classified genomic content measured in contig nucleotides.

Metric	PlaScope	PlasmidFinder	Platon
TP	2,884,199	1,776,553	2,745,897
TN	97,966,253	98,841,671	98,525,184
FP	1,309,315	433,897	750,384
FN	2,337,708	3,445,354	2,476,010

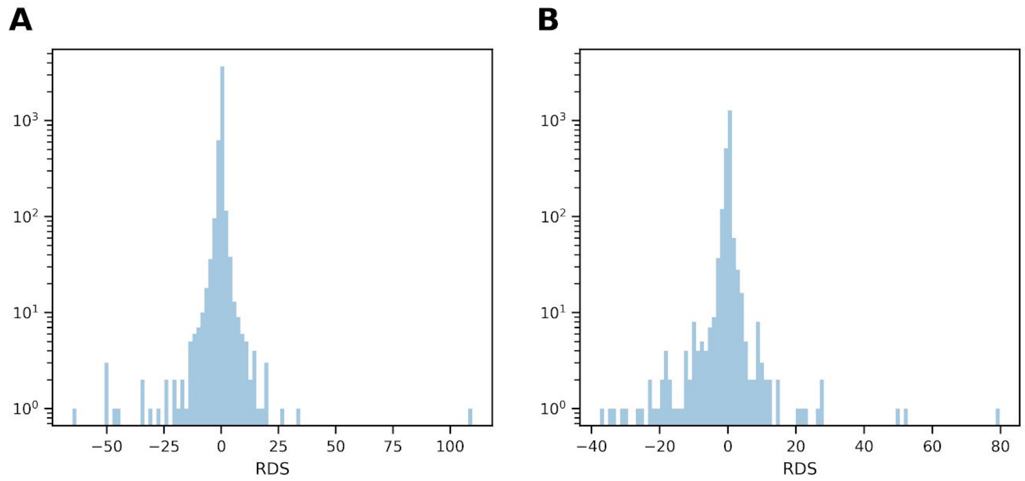

Supplementary Figure S1. Histogram of RDS values for (A) relaxase and (B) type 4-coupling proteins.

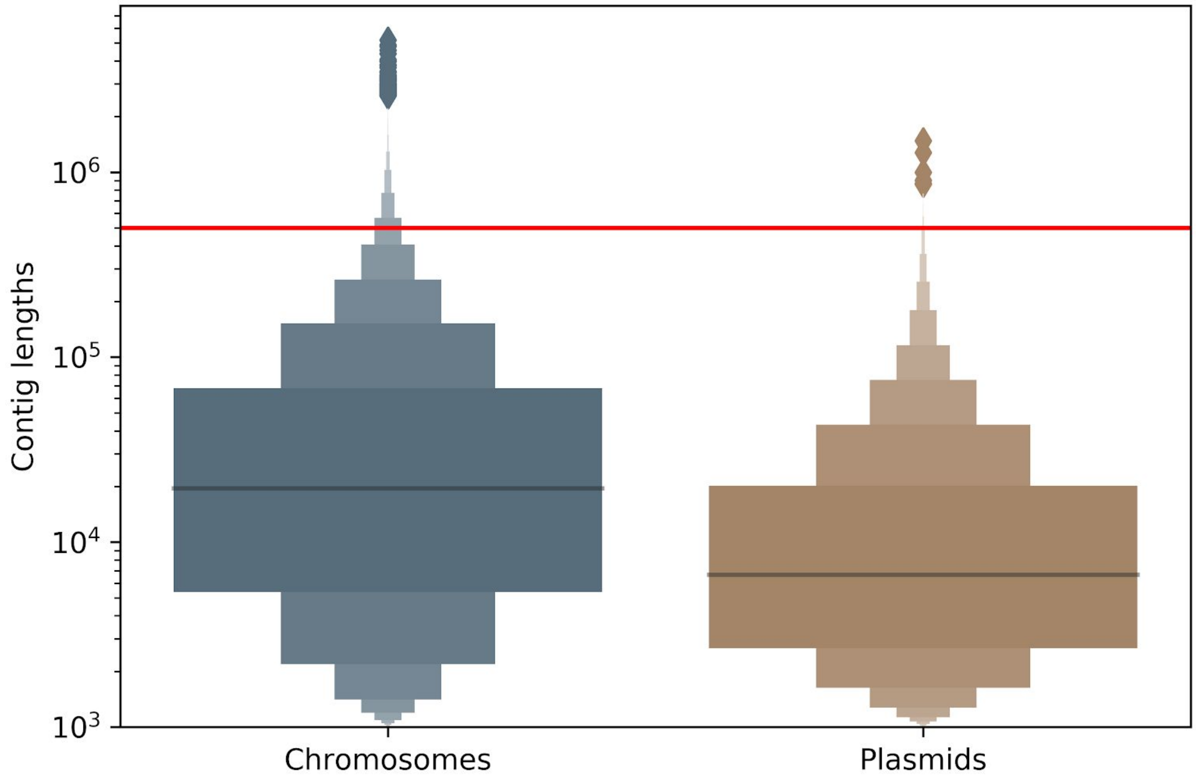

Supplementary Figure S2. Length distribution of chromosome and plasmid contigs resulted from simulated short-read assemblies. Outliers are shown as diamonds; horizontal red line: implemented contig length heuristic threshold (n=500,000 bp) as applied in the platon workflow.
